# Supplementary material for: COVID-19 mRNA vaccine induced antibody responses against three SARS-CoV-2 variants
Source: Nat Commun. 2021 Jun 28;12:3991. doi: 10.1038/s41467-021-24285-4 (PMC8239026; doi:10.1038/s41467-021-24285-4)
Supplement: Supplementary file 1 — Supplementary Information [file 41467_2021_24285_MOESM1_ESM.pdf]

Supplementary Information
for COVID-19 mRNA vaccine induced antibody responses and neutralizing antibodies against three SARS-CoV-2 variants

Pinja Jalkanen, Pekka Kolehmainen, Hanni K. Häkkinen, Moona Huttunen, Paula A. Tähtinen, Rickard Lundberg, Sari Maljanen, Arttu Reinholm, Sisko Tauriainen, Sari H. Pakkanen, Iris Levonen, Arttu Nousiainen, Taru Miller, Hanna Välimaa, Lauri Ivaska, Arja Pasternack, Rauno Naves, Olli Ritvos, Pamela Österlund, Suvi Kuivanen, Teemu Smura, Jussi Hepojoki, Olli Vapalahti, Johanna Lempainen, Laura Kakkola, Anu Kantele, Ilkka Julkunen

Contents

Supplementary Figures 1-3

Supplementary Table 1

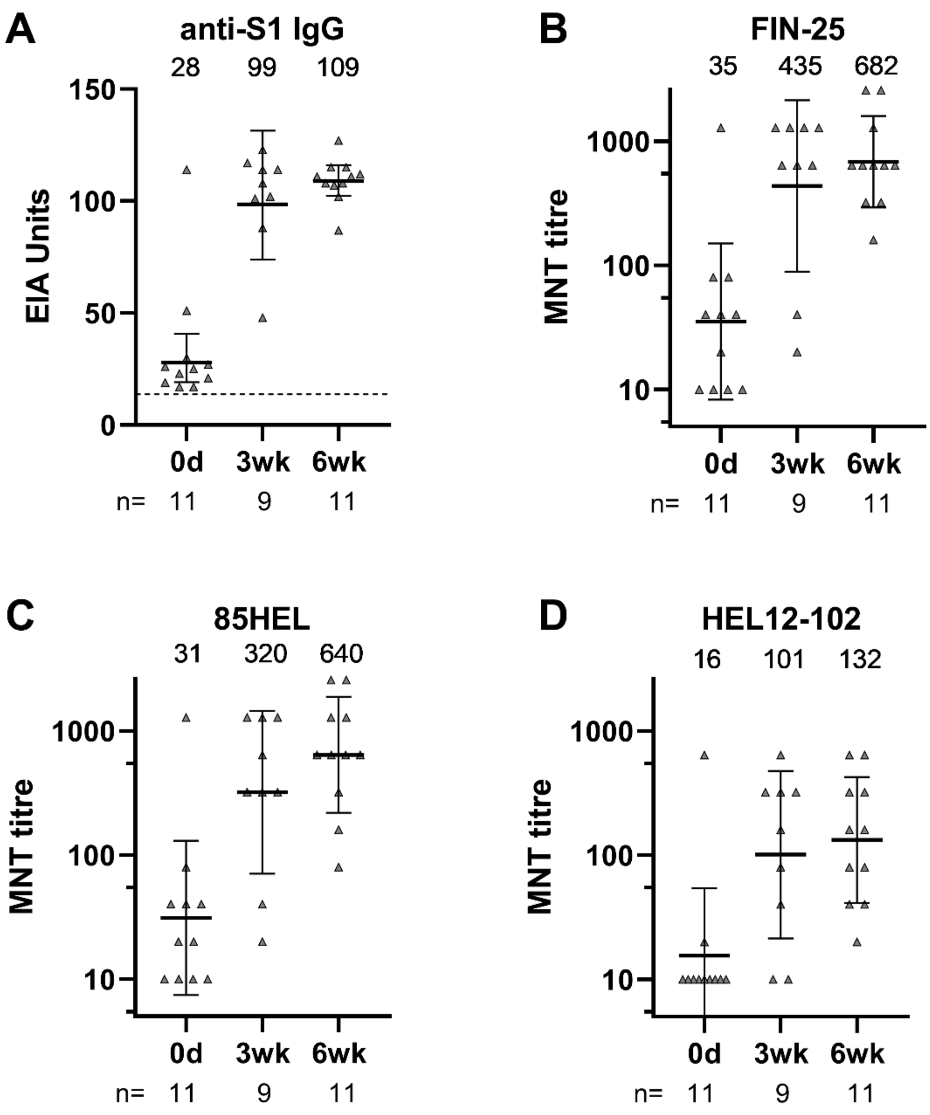

Supplementary Fig. 1 Antibody responses in BNT162b2 vaccinated health care workers with pre-existing anti-S1 IgG antibodies. A. Anti-S1 IgG antibody responses in 1:300 serum dilutions were determined with EIA. **B-D.** Neutralizing antibody titres against D614G variant FIN-25 (B), B.1.1.7 variant 85HEL (C) and B.1.351 variant HEL12-102 (D) determined with microneutralization test (MNT). Sequential serum samples were collected before vaccination (0d), and three (3wk) and six (6wk; 3 wk after the second vaccine dose) weeks after the first dose of the BNT162b2 vaccine. Geometric means are indicated on top of the graphs and data are presented as geometric means and geometric SDs. Dotted line (A) represents the cut-off value in IgG EIA. In MNT (B) samples with a titre value of <20 (considered negative) is given a value of 10. N refers to the number of serum specimens analysed.

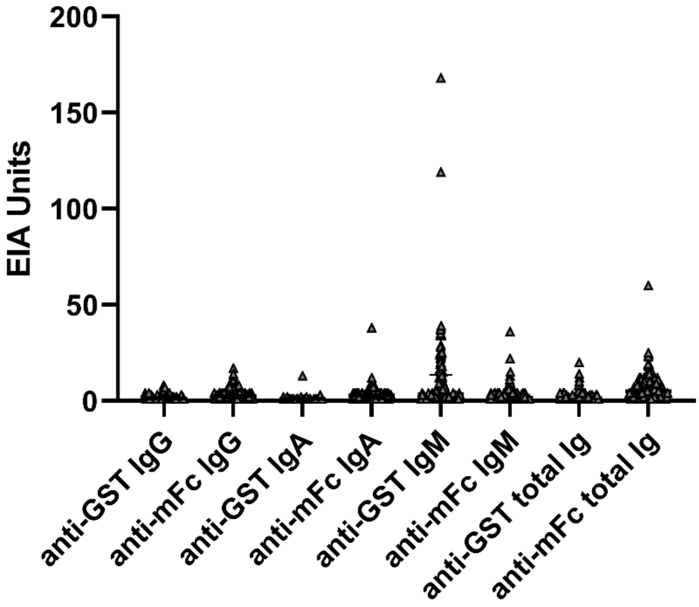

Supplementary Fig. 2 IgG, IgA, IgM and total Ig antibody responses against negative control antigens in serum samples collected from study participants before BNT162b2 vaccination (n=180). GST was used as a negative control antigen for recombinant SARS-CoV-2 nucleoprotein (N) and mouse promyostatin (ProMstn)-mFc(IgG2a)-6xhis protein, indicated as mFc, was used as a negative control for recombinant SARS-CoV-2 spike S1 domain. Data are presented as geometric means and geometric SDs.

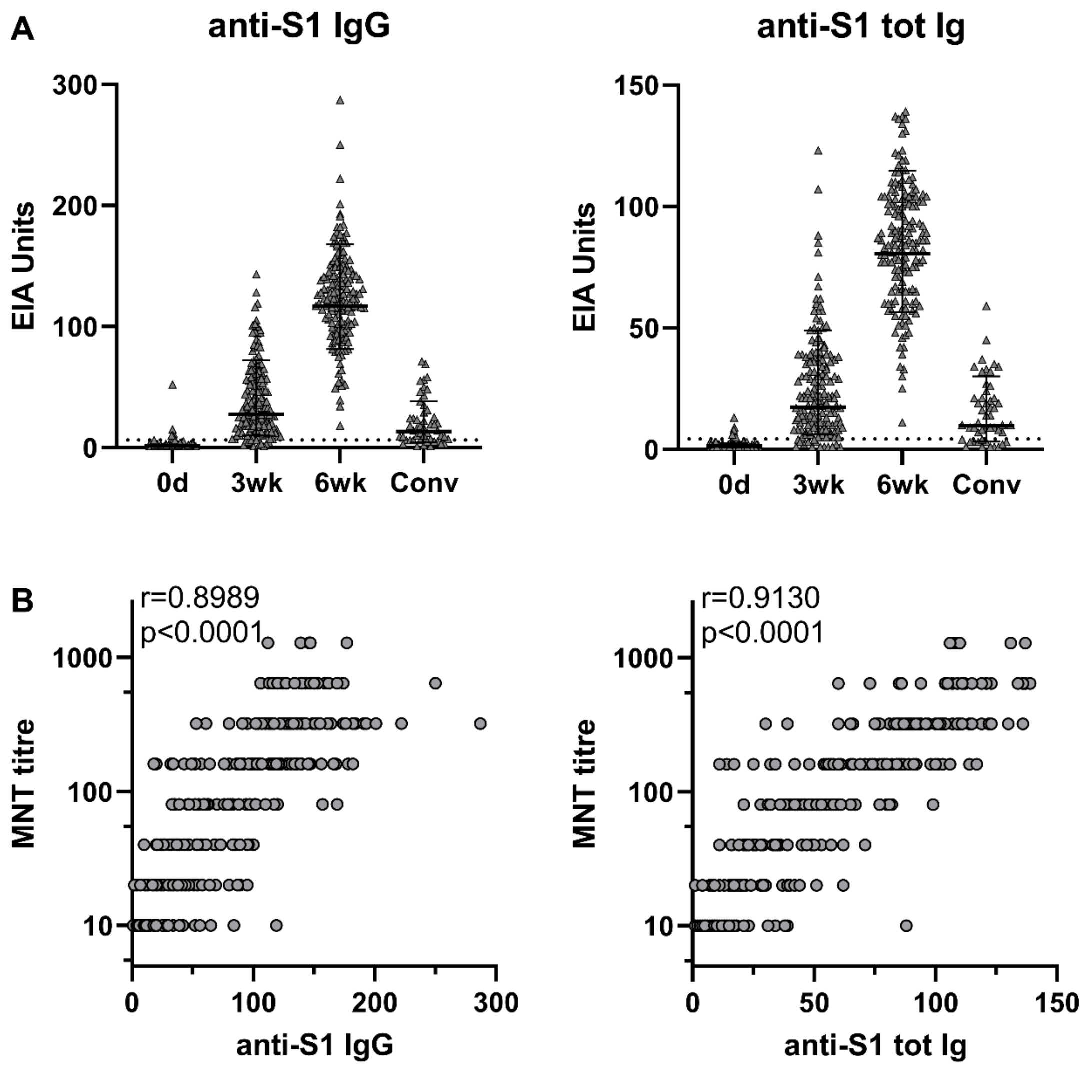

Supplementary Fig. 3 Anti-S1 antibody responses in BNT162b2 vaccinated health care workers determined in high serum dilutions. A. IgG and total Ig antibody levels were measured with S1-based EIA in 1:1000 serum dilution (n=159). Sequential serum samples were collected before vaccination (0d), and three (3wk) and six (6wk; 3 wk after the second vaccine dose) weeks after the first dose of the BNT162b2 vaccine. The data are represented as geometric means and geometric SDs. **B.** Correlation between anti-S1 IgG and total Ig responses in 1:1000 diluted sera and neutralization titres against FIN-25 virus isolate. Neutralization titres <20 were plotted as 10. Spearman's rank correlation coefficient (r) is indicated with two-tailed p-value.

Supplementary Table 1 Complete list of primer pools targeting SARS-CoV-2 used in the study.

Name	Sequence	Pool
NC_1_LEFT	ACCAACCAACTTTCGATCTCTTGT	1
NC_1_RIGHT	CGAGCATCCGAACGTTTGATGA	1
NC_2_LEFT	TCTTAAAGATGGCACTTGTGGCT	2
NC_2_RIGHT	ATGCACTCAAGAGGGTAGCCAT	2
NC_3_LEFT	AGTGGTGTTACCCGTGAACTCA	1
NC_3_RIGHT	CGTCTGCCATGAAGTTTCACCA	1
NC_4_LEFT	TCGATCTGTCTATCCAGTTGCGT	2
NC_4_RIGHT	AAGGTTGTCATTAAGACCTTCGGAAC	2
NC_5_LEFT	GGTTGCCATAACAAGTGTGCCT	1
NC_5_RIGHT	TAGCGGCCTTCTGTAAAACACG	1
NC_6_LEFT	CATTTGCATCAGAGGCTGCTCG	2
NC_6_RIGHT	AGAGTCAGCACACAAAGCCAAA	2
NC_7_LEFT	TGCTTGTGAAATTGTCGGTGGA	1
NC_7_RIGHT	AGTAACCTTTGTTGGTGCACCG	1
NC_8_LEFT	TGTTGCTCGAAATCAAAGACACAGA	2
NC_8_RIGHT	AGTGGCACCAAATTCCAAAGGT	2
NC_9_LEFT	TGAGGATGAAGAAGAAGGTGATTGTG	1
NC_9_RIGHT	TCCGCTTAAAACACAACTACCACC	1
NC_10_LEFT	AGGTGTTGCAGGAGCCTTAAAT	2
NC_10_RIGHT	TCTTGTTTTCTCTGTTCAACTGAAGGT	2
NC_11_LEFT	TGAAGAGTGAAAAGCAAGTTGAACAA	1
NC_11_RIGHT	AAGCACTGTCTTTGCCTCCTCT	1
NC_12_LEFT	CGAAAGCTTTGAGAAAAGTGCCA	2
NC_12_RIGHT	ACCGAGCAGCTTCTTCCAAATT	2
NC_13_LEFT	ACTGTAGCGTCACTTATCAACACAC	1
NC_13_RIGHT	CACAACTTGCGTGTGGAGGTTA	1
NC_14_LEFT	TCACCTTTGACAATCTTAAGACACTTCT	2
NC_14_RIGHT	GCACAAAAGTTAGCAGCTTCACC	2
NC_15_LEFT	GCCACTGCATTGTTAACACTCC	1
NC_15_RIGHT	AGCACCGTCTATGCAATACAAAGT	1
NC_16_LEFT	CAGTATGAACTTAAGCATGGTACATTTACT	2
NC_16_RIGHT	AGCCACCACATCACCATTTAAGT	2
NC_17_LEFT	CCATATCCAAACGCAAGCTTCG	1
NC_17_RIGHT	TGTGTGGCCAACCTCTTCTGTA	1
NC_18_LEFT	CCATACAGAAAGACGTTCTTGAGTGT	2
NC_18_RIGHT	GCCTCTAGACAAAATTTACCGACACT	2
NC_19_LEFT	AAACCGTGTTTGTACTAATTATATGCCTT	1
NC_19_RIGHT	TGCCAAAAACCACTCTGCAACT	1
NC_20_LEFT	GTGGTTTAGATTCTTTAGACACCTATCCT	2
NC_20_RIGHT	AGTTTGCAAAAGCCTTTACCTCCA	2
NC_21_LEFT	AACGTAATAGAGCAACAAGAGTCGA	1

NC_21_RIGHT	GCAACTTCCGCACTATCACCAA	1
NC_22_LEFT	TCATCTGCAAAATCAGCGTCTGT	2
NC_22_RIGHT	TGACTTTTTGCTACCTGCGCAT	2
NC_23_LEFT	AGAAGTTACTGGCGATAGTTGTAATAACT	1
NC_23_RIGHT	TAACTACCACCACGCTGGCTAA	1
NC_24_LEFT	AGGCTATTGATGGTGGTGTCAC	2
NC_24_RIGHT	AGCCATCCATGAGCACATAACG	2
NC_25_LEFT	TGGTAAGCCAGTACCATATTGTTATGA	1
NC_25_RIGHT	GGCAACTACATGACTGTATTCACCA	1
NC_26_LEFT	AGCATCTATAGTAGCTGGTGGTATTGT	2
NC_26_RIGHT	AGCACATCACTACGCAACTTTAGA	2
NC_27_LEFT	ACGTGTAGTCTTTAATGGTGTTTCCT	1
NC_27_RIGHT	AGCATGTCTTCAGAGGTGCAGA	1
NC_28_LEFT	TCCCATCTGGTAAAGTTGAGGGT	2
NC_28_RIGHT	ACCTTCTAAGTCTGTGCCAGCA	2
NC_29_LEFT	TGGTTCATGTGGTAGTGTTGGTT	1
NC_29_RIGHT	AACCAGTGGTGTGTACCCTTGA	1
NC_30_LEFT	GGACGTACCATATTGGGTAGTGC	2
NC_30_RIGHT	GTCCACACTCTCCTAGCACCAT	2
NC_31_LEFT	TGTCTGGTTTTAAGCTAAAAGACTGTGT	1
NC_31_RIGHT	TGCCACCAACACCCAACAATTT	1
NC_32_LEFT	CCGCTACTTTAGACTGACTCTTGG	2
NC_32_RIGHT	ATCACCATTAGCAACAGCCTGC	2
NC_33_LEFT	GGCAACCTTACAAGCTATAGCCT	1
NC_33_RIGHT	GGATTTCCCACAATGCTGATGC	1
NC_34_LEFT	ACCTCTTACAACAGCAGCCAAAC	2
NC_34_RIGHT	CCTACAAGGTGGTTCCAGTTCTG	2
NC_35_LEFT	GGAGGTAGGTTTGTACTTGCACTG	1
NC_35_RIGHT	CTATGTGGCAACGGCAGTACAG	1
NC_36_LEFT	ACACTGGTACTGGTCAGGCAAT	2
NC_36_RIGHT	CGTCCTTTTCTTGGAAGCGACA	2
NC_37_LEFT	ACAGGCACTAGTACTGATGTCGT	1
NC_37_RIGHT	AATACCAGCATTTCGCATGGCA	1
NC_38_LEFT	TGTAGAAAACCCAGATATATTACGCGT	2
NC_38_RIGHT	GTGGTCCAAAACTTGTAGGTGGG	2
NC_39_LEFT	TCAGACATACCACCCAAATTGTGT	1
NC_39_RIGHT	GCAGCATTACCATCCTGAGCAA	1
NC_40_LEFT	ACAAAGACTTCTATGACTTTGCTGTGT	2
NC_40_RIGHT	CTCCTCTAGTGGCGGCTATTGA	2
NC_41_LEFT	TGCAAAGAATAGAGCTCGCACC	1
NC_41_RIGHT	TGCATTAACATTGGCCGTGACA	1
NC_42_LEFT	TGGCGGTTCACTATATGTTAAACCA	2
NC_42_RIGHT	GGCCCCTAGGATTCTTGATGGA	2
NC_43_LEFT	ACTAAAGGACCTCATGAATTTTGCTCT	1
NC_43_RIGHT	TGGTCTACGTATGCAAGCACCA	1

NC_44_LEFT	TGAGGCTATGTACACACCGCAT	2
NC_44_RIGHT	CTCTGTCAGACAGCACTTCACG	2
NC_45_LEFT	TCAAGCTTTTTGCAGCAGAAACG	1
NC_45_RIGHT	TGCGAGCAGAAGGGTAGTAGAG	1
NC_46_LEFT	TGCAAATTATCAAAAGGTTGGTATGCA	2
NC_46_RIGHT	CCGAGGAACATGTCTGGACCTA	2
NC_47_LEFT	GCACCACGCACATTGCTAACTA	1
NC_47_RIGHT	ACAAGAGTGAGCTGTTTCAGTGG	1
NC_48_LEFT	TGCTGTAGCCTCAAAGATTTTGGG	2
NC_48_RIGHT	GCTTCTTCGCGGGTGATAAACA	2
NC_49_LEFT	TGGCATACCTAAGGACATGACCT	1
NC_49_RIGHT	CATAGACAACAGGTGCGCTCAG	1
NC_50_LEFT	CTCTCTGACAGAGTCGTATTTGTCTT	2
NC_50_RIGHT	ACCAATGTCGTGAAGAACTGGG	2
NC_51_LEFT	TGATGAACTGAAGATTAATGCGGCT	1
NC_51_RIGHT	AGACTCACATGGACTGTCAGAGT	1
NC_52_LEFT	ATAAACATGCATTCCACACACCAG	2
NC_52_RIGHT	TGGTACTGGTTTAATGTTGCGCT	2
NC_53_LEFT	ACTGTTTACACAAAAGTTGATGGTGT	1
NC_53_RIGHT	TGTTGGACAACACCATCAACTTTCT	1
NC_54_LEFT	CCATCTGTAGGTCCCAAACAAGC	2
NC_54_RIGHT	TCACTTTGACAACCTTAGAAACTACAGA	2
NC_55_LEFT	AGATGCGCAAACAGGTTCATCT	1
NC_55_RIGHT	GATCTGAATCGACAAGCAGCGT	1
NC_56_LEFT	TGAGAGTTATACATTTTGGTGCTGGT	2
NC_56_RIGHT	CTATTTGTTCGCGTGGTTTGCC	2
NC_57_LEFT	GCATGGTGGACAGCCTTTGTTA	1
NC_57_RIGHT	CAGGGTAATAAACACCACGTGTGA	1
NC_58_LEFT	TGTTTTATTGCCACTAGTCTCTAGTCA	2
NC_58_RIGHT	AGGTCCATAAGAAAAGGCTGAGAGA	2
NC_59_LEFT	CCACAAAAACAACAAAAGTTGGATGG	1
NC_59_RIGHT	TCTGAGAGAGGGTCAAGTGCAC	1
NC_60_LEFT	GTGCTGCAGCTTATTATGTGGGT	2
NC_60_RIGHT	TTCCAAGCTATAACGCAGCCTG	2
NC_61_LEFT	TGAAGTCAGACAAATCGCTCCAG	1
NC_61_RIGHT	ACGGACAGCATCAGTAGTGTCA	1
NC_62_LEFT	TGGTTTAACAGGCACAGGTGTT	2
NC_62_RIGHT	GCACCAAGTGACATAGTGTAGGC	2
NC_63_LEFT	ACCCATTGGTGCAGGTATATGC	1
NC_63_RIGHT	TGATGAAGCCAGCATCTGCAAG	1
NC_64_LEFT	AGTCAAACAAATTTACAAAACACCACCA	2
NC_64_RIGHT	GCTTGTGCATTTTGGTTGACCA	2
NC_65_LEFT	GCCAACCAATTTAATAGTGCTATTGGC	1
NC_65_RIGHT	CATGACAAATGGCAGGAGCAGT	1
NC_66_LEFT	CTTCCCTCAGTCAGCACCTCAT	2

NC_66_RIGHT	CAAGCCAGCTATAAAACCTAGCCA	2
NC_67_LEFT	TGAGGTTGCCAAGAATTTAAATGAATCT	1
NC_67_RIGHT	TGGAGAGTGCTAGTTGCCATCT	1
NC_68_LEFT	TTCGGATGGCTTATTGTTGGCG	2
NC_68_RIGHT	ACACAGTCTTTTACTCCAGATTCCC	2
NC_69_LEFT	GTCATTACTTCAGGTGATGGCACA	1
NC_69_RIGHT	ACACAATCGAAGCGCAGTAAGG	1
NC_70_LEFT	CGTTTCGGAAGAGACAGGTACG	2
NC_70_RIGHT	GCAATTCCACCGGTGATCCAAT	2
NC_71_LEFT	CTATGCCAACAGGAATAGGTTTTTGT	1
NC_71_RIGHT	TGCCAATCCTGTAGCGACTGTA	1
NC_72_LEFT	CTGTTGCTACATCACGAACGCT	2
NC_72_RIGHT	TGCCCTCGTATGTTCCAGAAGA	2
NC_73_LEFT	CTTGGCACTGATAACACTCGCT	1
NC_73_RIGHT	TGTTCGTTTAGGCGTGACAAGT	1
NC_74_LEFT	TTTGTGCTTTTTAGCCTTTCTGCT	2
NC_74_RIGHT	TGCATTTCGCTGATTTTGGGGT	2
NC_75_LEFT	ATGAAGACTTTTTAGAGTATCATGACGTTC	1
NC_75_RIGHT	GATGCCGTCTTTGTTAGCACCA	1
NC_76_LEFT	CGTGGTGGTGACGGTAAAATGA	2
NC_76_RIGHT	GCTTCTTAGAAGCCTCAGCAGC	2
NC_77_LEFT	TGCTTGACAGATTGAACCAGCT	1
NC_77_RIGHT	TCTGCAGCAGGAAGAAGAGTCA	1
NC_78_LEFT	ACATTCCCACCAACAGAGCCTA	2
NC_78_RIGHT	TTAGGGCTCTTCCATATAGGCAGC	2
