# Supplementary material for: COVID-19 mRNA vaccine induced antibody responses against three SARS-CoV-2 variants
Source: Nat Commun. 2021 Jun 28;12:3991. doi: 10.1038/s41467-021-24285-4 (PMC8239026; doi:10.1038/s41467-021-24285-4)
Supplement: Supplementary file 3 — Reporting Summary [file 41467_2021_24285_MOESM3_ESM.pdf]

nature research

Corresponding author(s): Pinja Jalkanen and Ilkka Julkunen

Last updated by author(s): Jun 5, 2021

Reporting Summary

Nature Research wishes to improve the reproducibility of the work that we publish. This form provides structure for consistency and transparency in reporting. For further information on Nature Research policies, see our Editorial Policies and the Editorial Policy Checklist.

Statistics

For all statistical analyses, confirm that the following items are present in the figure legend, table legend, main text, or Methods section.

n/a	Confirmed	
☐	☒	The exact sample size (*n*) for each experimental group/condition, given as a discrete number and unit of measurement
☐	☒	A statement on whether measurements were taken from distinct samples or whether the same sample was measured repeatedly
☐	☒	The statistical test(s) used AND whether they are one- or two-sided<br>*Only common tests should be described solely by name; describe more complex techniques in the Methods section.*
☐	☒	A description of all covariates tested
☐	☒	A description of any assumptions or corrections, such as tests of normality and adjustment for multiple comparisons
☐	☒	A full description of the statistical parameters including central tendency (e.g. means) or other basic estimates (e.g. regression coefficient) AND variation (e.g. standard deviation) or associated estimates of uncertainty (e.g. confidence intervals)
☐	☒	For null hypothesis testing, the test statistic (e.g. F, t, r) with confidence intervals, effect sizes, degrees of freedom and P value noted<br>*Give P values as exact values whenever suitable.*
☒	☐	For Bayesian analysis, information on the choice of priors and Markov chain Monte Carlo settings
☒	☐	For hierarchical and complex designs, identification of the appropriate level for tests and full reporting of outcomes
☐	☒	Estimates of effect sizes (e.g. Cohen's *d*, Pearson's *r*), indicating how they were calculated

Our web collection on statistics for biologists contains articles on many of the points above.

Software and code

Policy information about availability of computer code

Data collection	No software was used.
Data analysis	Excel 2016 (Microsoft), Prism v8 (GraphPad Software), PrimalScheme, Trimmomatic version 0.39 (Bolger, A. M., Lohse, M., & Usadel, B. (2014). Trimmomatic: A flexible trimmer for Illumina Sequence Data. Bioinformatics), BWA-MEM algorithm, SAMTools v1.847 (Li, H. et al. The Sequence Alignment/Map format and SAMtools. Bioinformatics) and YASARA (available at http://www.yasara.org/).

For manuscripts utilizing custom algorithms or software that are central to the research but not yet described in published literature, software must be made available to editors and reviewers. We strongly encourage code deposition in a community repository (e.g. GitHub). See the Nature Research guidelines for submitting code & software for further information.

Data

Policy information about availability of data

All manuscripts must include a data availability statement. This statement should provide the following information, where applicable:

- Accession codes, unique identifiers, or web links for publicly available datasets
- A list of figures that have associated raw data
- A description of any restrictions on data availability

All data are available upon request from the corresponding authors. Source data are provided with this paper. The SARS-CoV-2 sequence data generated in this study have been deposited in the Genbank database under accession codes MW717675 [https://www.ncbi.nlm.nih.gov/nuccore/MW717676], MW717676 [https://www.ncbi.nlm.nih.gov/nuccore/MW717676], MW717677 [https://www.ncbi.nlm.nih.gov/nuccore/MW717677] and MW717678 [https://www.ncbi.nlm.nih.gov/nuccore/MW717678].

Field-specific reporting

Please select the one below that is the best fit for your research. If you are not sure, read the appropriate sections before making your selection.

☒ Life sciences ☐ Behavioural & social sciences ☐ Ecological, evolutionary & environmental sciences

For a reference copy of the document with all sections, see nature.com/documents/nr-reporting-summary-flat.pdf

Life sciences study design

All studies must disclose on these points even when the disclosure is negative.

Sample size	Due to the exploratory nature of the study, no sample-size calculation was performed. The study included 536 serum samples from 180 vaccinated individuals, 50 serum samples from 50 convalescent phase patients and 20 serum samples from 20 individuals prior to the pandemic. All available samples were analyzed.
Data exclusions	No data was excluded.
Replication	All serum samples were analyzed in duplicates.
Randomization	All the available samples were tested in this study and no randomization was performed. The study is observational and thus covariates were not controlled and participants were included without any exclusion criteria.
Blinding	The study was not blinded for group allocation and principally all study participants were included into the study in an open label principle. The recruitment to the study was based on the time of vaccination. The first 180 participants who received their two vaccine doses were included in the study.

Reporting for specific materials, systems and methods

We require information from authors about some types of materials, experimental systems and methods used in many studies. Here, indicate whether each material, system or method listed is relevant to your study. If you are not sure if a list item applies to your research, read the appropriate section before selecting a response.

Materials & experimental systems

n/a	Involved in the study	
☐	☒	Antibodies
☐	☒	Eukaryotic cell lines
☒	☐	Palaeontology and archaeology
☒	☐	Animals and other organisms
☐	☒	Human research participants
☐	☒	Clinical data
☒	☐	Dual use research of concern

Methods

n/a	Involved in the study	
☒	☐	ChIP-seq
☒	☐	Flow cytometry
☒	☐	MRI-based neuroimaging

Antibodies

Antibodies used	Rabbit anti-human IgG HRP (Dako A/S, P021402-2), Goat anti-human IgA (a-chain) HRP (Invitrogen, A18787), Rabbit anti-human IgM HRP (Dako A/S, P021502-2) and Goat anti-human IgG+IgA+IgM (H&L) HRP (Abcam, ab102420).
Validation	Validation of HRP conjugated antibodies is available through manufacturers' website. All HRP conjugated antibodies are tested by the manufacturers for specificity by immunoelectrophoresis and ELISA. Intended use is the determination of human antibodies in ELISA. For our in-house EIA, HRP conjugates were titrated to find the optimal concentration i.e. high sensitivity and low background.

Eukaryotic cell lines

Policy information about cell lines

Cell line source(s)	VeroE6 cells (ATCC® CRL-1586) were obtained from the ATCC. VeroE6-TMPRSS2-H10 cells were obtained from Dr. Jussi Hepojoki (Rusanen, J. et al. A generic, scalable, and rapid TR-FRET –based assay for SARS-CoV-2 antigen 473 detection. medRxiv). HEK293F cells were obtained from Thermo Fisher (R79007) and Sf9 cells were obtained from ATCC (ATCC® CRL-1711).
Authentication	VeroE6 and Sf9 cells were authenticated by ATCC, and HEK293F cells by Thermo Fisher. VeroE6-TMPRSS2-H10 cells were authenticated in Western Blot with mouse monoclonal anti-V5 epitope tag antibody by Dr. Jussi Hepojoki (For more

	information and results see: Rusanen, J. et al. A Generic, Scalable, and Rapid Time-Resolved Förster Resonance Energy Transfer-Based Assay for Antigen Detection—SARS-CoV-2 as a Proof of Concept. mBio (2021)).
Mycoplasma contamination	All cell lines were tested negative for mycoplasma.
Commonly misidentified lines (See ICLAC register)	None

Human research participants

Policy information about studies involving human research participants

Population characteristics	Study participants (n=180) were recruited among healthcare personnel of Turku University Hospital and Helsinki University Hospital prior to receiving vaccine as part of hospital occupational health care. The characteristics of participants were not pre-established. Study participants were 20-65 years old and 83% were female. Non-hospitalized individuals with confirmed COVID-19 were invited to participate. COVID-19 patients were 19-93 years old and 66% were female. Randomly selected diagnostic serum specimens (n=20) were included as negative controls and the samples were fully de-identified.
Recruitment	The study participants were recruited among health care personnel at Turku University Hospital and Helsinki University Hospital. According to national vaccination priority principles, health care workers treating COVID-19 patients and laboratory personnel involved in COVID-19 diagnostics were vaccinated first and the vaccination programme started on the 28th of December, 2021. Hospital occupational health care was responsible for organizing the vaccination campaign. The characteristics of participants were not pre-established and all participants were voluntarily included into the study without any exclusion criteria. Age and gender distribution of participants represents well the study group and self-selection bias is not likely to impact the results of observational study. Non-hospitalized individuals with a confirmed COVID-19 infection were included as patients. All vaccinees and COVID-19 patients gave their written informed consent for the study. Randomly selected pseudonymed serum specimens from the routine diagnostics of Turku University Hospital Clinical microbiology unit were collected in early 2019 and included as negative controls. Written informed consent was not recuired for these specimens, since they were de-identified. The recuitment of the vaccinee and patient sera and control serum specimens was done according to valid ethical permissions and sample collection permissions of the respective hospital districts.
Ethics oversight	The cohorts were approved by institutional review board of each health care district. At enrollment a written informed consent was collected from all participants.

Note that full information on the approval of the study protocol must also be provided in the manuscript.

Clinical data

Policy information about clinical studies

All manuscripts should comply with the ICMJE guidelines for publication of clinical research and a completed CONSORT checklist must be included with all submissions.

Clinical trial registration	The used Pfizer-BioNTech vaccine was an EMA-accepted licensed vaccine and the vaccine was used according to national guidelines and given by the hospital district occupational health care system. The study is thus observational and the authors were not responsible for giving the vaccine nor for the potential side effects of the vaccine. Since the data collected is not used for vaccine registration purposes nor experimental, according to national guidelines no clinical trial registration is required.
Study protocol	The characteristics of this observational study has been described in Materials and Methods and no separate Study protocol is included.
Data collection	The Data collection started in December 2020 and the collection of the samples continues for up to 5 years from the start. The presently valid ethical and health district permissions allow this long follow-up time.
Outcomes	The study is an observational study on immune responses induced by a European Union licensed COVID-19 vaccine which is given to the participants as part of a national vaccination programme. The study thus does not include any predefined outcomes.
